# Relationships between self-perceived and clinical expression of pain and function differ based on the underlying pathology of the human hip

**DOI:** 10.1186/s12891-023-06768-1

**Published:** 2023-08-07

**Authors:** Brandon Nunley, Edward P. Mulligan, Avneesh Chhabra, Nicholas P. Fey, Joel Wells

**Affiliations:** 1 Department of Biomedical Engineering, The University of Texas at Austin, Austin, TX USA; 2 School of Medicine, Tufts University, Boston, MA USA; 3 Department of Radiology, UT Southwestern Medical Center, Dallas, TX USA; 4 Walker Department of Mechanical Engineering, The University of Texas at Austin, Austin, TX USA; 5 Department of Orthopedic Surgery, Baylor Scott & White Medical Center, 301 N. Washington Ave, Dallas, TX 75246 USA

**Keywords:** Femoroacetabular impingement syndrome, Developmental dysplasia of the hip, Patient-reported measures, Correlation analysis, Hip pain, Physical activity

## Abstract

**Background:**

Patient-reported outcomes are commonly used to assess patient symptoms. The effect of specific hip pathology on relationships between perceived and objectively measured symptoms remains unclear. The purpose of this study was to evaluate differences of function and pain in patients with FAIS and DDH, to assess the correlation between perceived and objective function, and to determine the influence of pain on measures of function.

**Methods:**

This prospective cross-sectional study included 35 pre-operative patients (60% female) with femoroacetabular impingement syndrome (FAIS) and 37 pre-operative patients (92% female) with developmental dysplasia of the hip (DDH). Objectively measured function (6-min walk [6MWT], single leg hop [SLHT], Biodex sway [BST], hip abduction strength [HABST], and STAR excursion balance reach [STAR] tests), patient-reported function (UCLA Activity, Hip Outcome Score [HOS], Short Form 12 [SF-12], and Hip Disability and Osteoarthritis Outcome Score [HOOS]), and patient-reported pain (HOOS Pain, visual analogue scale (VAS), and a pain location scale) were collected during a pre-surgical clinic visit. Between-group comparisons of patient scores were performed using Wilcoxon Rank-Sum tests. Within-group correlations were analyzed using Spearman’s rank correlation coefficients. Statistical correlation strength was defined as low (*r* =  ± 0.1–0.3), moderate (*r* =  ± 0.3–0.5) and strong (*r* >  ± 0.5).

**Results:**

Patients with DDH reported greater pain and lower function compared to patients with FAIS. 6MWT distance was moderately-to-strongly correlated with a number of patient-reported measures of function (FAIS: *r* = 0.37 to 0.62, DDH: *r* = 0.36 to 0.55). Additionally, in patients with DDH, SLHT distance was well correlated with patient reported function (*r* = 0.37 to 0.60). Correlations between patient-reported pain and objectively measured function were sparse in both patient groups. In patients with FAIS, only 6MWT distance and HOOS Pain (*r* = -0.53) were significantly correlated. In patients with DDH, 6MWT distance was significantly correlated with VAS Average (*r* = -0.52) and Best (*r* = -0.53) pain.

**Conclusion:**

Pain is greater and function is lower in patients with DDH compared to patients with FAIS. Moreover, the relationship between pain and function differs between patient groups. Understanding these differences is valuable for informing treatment decisions. We recommend these insights be incorporated within the clinical continuum of care, particularly during evaluation and selection of surgical and therapeutic interventions.

**Supplementary Information:**

The online version contains supplementary material available at 10.1186/s12891-023-06768-1.

## Background

Femoroacetabular impingement syndrome (FAIS) and developmental dysplasia of the hip (DDH) are common causes of hip pain and impaired function in young adults that may lead to premature development of hip osteoarthritis [1–4]. FAIS is characterized by recurrent abutment of the femoral head-neck junction and acetabular rim during near-terminal joint articulation [5]. Morphologic causes of the dynamic pathomechanics of FAIS are excessive acetabular coverage (i.e., pincer morphology) and/or asphericity of the femoral head (i.e., cam morphology) [6]. DDH is characterized by a shallow acetabulum and lateralized hip joint center [7]. Although the pathomechanics of FAIS and DDH are different, both can limit the quality of life and cause hip pain.

Impaired physical function is associated with both FAIS and DDH [5, 8]; however, the specific mechanism by which aberrant hip morphology translates to impaired function is different between the two pathologies. For FAIS, recent findings suggest that altered biomechanics observed during level walking may be a protective mechanism [9]. In addition, although the hip range-of-motion (ROM) demands of level walking are unlikely to induce impingement, these patients tend to avoid excessive hip articulation as a response to previously experienced hip pain [10]. Thus, for patients with FAIS, reduced performance during less demanding tasks may be more attributable to psychological impediments rather than biomechanically impaired function. Patients with FAIS also demonstrate altered kinematics and kinetics during tasks with high hip ROM demands, such as squatting and stair climbing [11, 12]. By contrast in DDH, a shallow acetabulum and lateralized hip joint center result in lower torque generating capacity of the hip abductors and higher joint reaction forces. As a result, the hip joint may be destabilized [13, 14]. This localized mechanical effect manifests in broad biomechanical alterations, such as increased kinematic variability during ambulation and lower peak hip flexion during single-leg squatting compared to healthy individuals [15, 16].

Quantifying functional decrements in hip patients is important for treatment planning. Patient outcomes can be grouped into objectively measured function (OBJ-F), patient-reported outcome measures of function (PROM-F), and patient-reported outcome measures of pain (PROM-P). Treating physicians rely on PROM-F and PROM-P to assess symptoms and track post-surgery improvements. Numerous studies have examined the relationship between perceived and true function in patients who have undergone total hip and knee replacement [17–25], but less is known for patients with FAIS and DDH. Scott et al [26] demonstrated strong correlation between PROM-F and OBJ-F in patients with DDH, suggesting that such patients have an accurate perception of their own abilities that is not skewed by perceived limitations. As a secondary aim, the authors determined that patient-reported pain was not well correlated with measures of function in patients with DDH. By contrast, the psychological pain-avoidance mechanisms theorized for patients with FAIS may limit the agreement between perceived and true function [9]. That is, patients with FAIS may inaccurately estimate their level of functional impairment. The lack of knowledge of potential discrepancies between patient measurement modalities has been noted previously [27–29]. Improving our understanding of these relationships will provide insight into the pathophysiology of FAIS and DDH while informing the optimal use of patient data instruments.

The purpose of this study was (1) to evaluate differences of function and pain in patients with FAIS and DDH, (2) to assess the correlation between perceived and true functional ability, and (3) to determine the influence of pain on measures of function. We hypothesized that patients with DDH would report higher pain and perform worse on functional tasks that demand hip power and stability compared to patients with FAIS, that patients with FAIS would exhibit weaker correlation between OBJ-F and PROM-F compared to patients with DDH, and that, in both patient groups, PROM-P would be weakly correlated with OBJ-F compared to PROM-F.

## Materials and methods

### Patients and study design

This prospective cross-sectional study of patients with FAIS and patients with DDH was approved by the University of Texas Southwestern Institutional Review Board and completed during a 33-month time period. Patients scheduled for future hip preservation surgery at our institution were eligible for inclusion. All included patients were diagnosed by a fellowship-trained hip preservation orthopedic surgeon [30, 31]. Patients with symptomatic DDH, radiographic evidence of femoral head uncovering, and a lateral center–edge angle (LCEA) of < 25º were offered treatment with periacetabular osteotomy. Patients with symptomatic FAIS, radiographic evidence of FAIS CAM or Pincer, an alpha angle of > 65º, an LCEA of > 38º, head and neck offset deformity, and positive impingement sign were offered treatment with hip arthroscopy or surgical hip dislocation. Details of our radiographic procedure have been described previously by Wells et al [30]. The LCEA, Tönnis angle, joint space width, Tönnis grade, and congruency were measured on standing anteroposterior radiographs. The ACEA was measured on false-profile radiographs. Alpha angle was measured on Dunn and frog-leg radiographs. Exclusion criteria were onset of osteoarthritis or a history of previous osteotomy or arthroplasty in either hip. Written consent was obtained from all patients included in this study.

### Instrumentation

Questionnaire data, which have previously been validated [32–37], were collected during a pre-surgical clinic visit. Objective measures of function were also collected pre-operatively under the supervision of a single licensed physical therapist with 8 years of experience. Variables were grouped into OBJ-F, PROM-F, and PROM-P. OBJ-F instrumentation consisted of the 6-min walk test (6MWT), single leg hop test (SLHT), Biodex sway test (BST), hip abduction strength test (HABST), and STAR excursion balance reach tests (STAR) in multiple directions. The HABST was performed while side-lying with the testing limb in end-range extension and 10–20 degrees of abduction. The dynamometer was held proximal to the lateral malleolus while instruction was provided to abduct as hard as possible. For tests performed unilaterally, only scores for the limb indicated for surgery were included. PROM-F consisted of UCLA Activity scale, Hip Outcome Score (HOS) activities of daily living (ADL) and Sport subscales, Short Form 12 (SF-12) Physical Activity subscale, and the Hip Disability and Osteoarthritis Outcome Score (HOOS) ADL and Sport subscales. For PROM-P, the following were included: HOOS Pain subscale, visual analogue scale (VAS), and an anatomical pain location scale with the following body locations: groin, anterior thigh, knee, low back, buttock, posterior thigh, trochanter, and lateral thigh. The anatomical pain location scale was previously described by Nam et al [38]. The scores for frequency and intensity of pain were multiplied together as an aggregate measure of pain at each body location.

### Statistical methods

Statistical analyses were carried out using a custom Python script which included with the Scikit-learn and NumPy packages. Patient demographics were compared using independent two-sample t-tests, except for biological sex which was compared using a chi-square test. Between-group comparisons of patient scores were performed using Wilcoxon Rank-Sum tests. Additionally, the rank-biserial correlation, a measure of effect size commonly reported alongside the Wilcoxon Rank-Sum test, was computed. Within-group relationships between pairs of outcome variables were analyzed using Spearman’s rank correlation coefficients. The level of significance for between-group comparisons was set at α = 0.05. To address the problem of multiple comparisons that occurs during correlation of a large number of outcomes, the Benjamini–Hochberg procedure was performed with the false discovery rate set at Q = 0.05. Strength of correlations was defined as low (*r* =  ± 0.1–0.3), moderate (*r* =  ± 0.3–0.5) and strong (*r* >  ± 0.5) [39]. For the correlation analysis, the HOOS Pain and BST scales were inverted so that higher scores always indicate higher pain and function.

## Results

### Patient demographics

Forty-two patients with FAIS and 39 patients with DDH were approached for participation in the current study. Of the 42 patients with FAIS, seven were excluded due to previous osteotomy. Of the 42 patients with DDH, three were excluded due to previous osteotomy, one was excluded due to previous contralateral total hip replacement, and one was excluded due to onset of osteoarthritis. Our included sample of 35 patients with FAIS and 37 patients with DDH were 60% and 92% female, respectively (*p* = 0.001). The mean age was 33.46 years for patients with FAIS (SD: 11.89; range: 16**–**56) and 25.54 years for patients with DDH (SD: 6.24; range: 17**–**37). The patients with FAIS were significantly older than the patients with DDH (*p* < 0.001; CI: 3.49 to 12.35). Mean body mass index was not significantly different (*p* = 0.907; CI: -2.08 to 2.73) between the patients with FAIS (mean: 26.19 kg/m^2^, SD: 4.93) and patients with DDH (mean: 25.87 kg/m^2^, SD: 5.27). Radiographic characteristics of these patients are presented in Table 1.

**Table 1:** Radiographic measurements of patients

Characteristics	FAIS (*n* = 35)	DDH (*n* = 37)
Tönnis Angle (°)	1.3 (5.6)	14.2 (7.2)
LCEA (°)	30.0 (10.6)	12.8 (7.1)
ACEA (°)	34.1 (11.3)	15.1 (10.6)
Alpha Frog Angle (°)	61.8 (9.7)	68.4 (18.7)
Alpha Dunn Angle (°)	64.9 (9.9)	74.1 (18.4)
Joint Space Width (mm)	5.6 (7.2)	5.1 (0.7)
Tönnis grade, n (%)		
0	29 (83%)	34 (92%)
1	6 (17%)	3 (8%)
Congruency, n (%)		
Excellent	32 (91%)	31 (84%)
Good	3 (9%)	5 (14%)
Fair	0 (0%)	1 (3%)
Crossover Sign, n (%)		
Yes	11 (31%)	7 (19%)
No	24 (69%)	30 (81%)
Ischial Spine Sign, n (%)		
Yes	8 (23%)	6 (16%)
No	27 (77%)	31 (84%)
Posterior Wall Sign, n (%)		
Yes	15 (43%)	30 (81%)
No	20 (57%)	7 (19%)

Summary statistics are presented as *Mean (SD*) unless otherwise indicated

### Direct comparison of outcomes between patient groups

No differences in objectively measured function were detected between patients with FAIS and DDH (Table 2). Patients with DDH indicated significantly lower activity and physical function in ADL and Sport domains compared to patients with FAIS as indicated by UCLA Activity (*p*** = **0.025), HOS ADL (*p*** = **0.013), SF-12 Physical Function (*p*** = **0.021), and HOOS Sport (*p*** = **0.030) scores (Table 3). Patients with DDH also reported significantly higher levels of pain compared to patients with FAI as indicated by HOOS Pain (*p*** = **0.012) score (Table 4).

**Table 2:** Objectively measured function scores

Variables	FAIS	DDH	U	*p*	*r*_rb_
6MWT Distance (m)	510.75 (138.35)	469.60 (107.10)	823	**0.049**	-0.271
SLHT (cm)	114.43 (38.84)	101.25 (33.72)	717	0.437	-0.107
BST Eyes Open	1.67 (0.83)	1.58 (0.65)	659.5	0.897	-0.019
BST Eyes Closed	3.48 (1.05)	3.32 (0.73)	646	0.991	0.002
Hip Abduction Strength (N)	65.95 (25.97)	54.22 (5.45)	841.5	**0.029**	-0.300
Star Excursion Balance					
Anterior (cm)	62.43 (10.18)	63.08 (24.24)	596.5	0.569	0.079
Lateral (cm)	76.71 (11.17)	75.55 (17.10)	620	0.761	0.042
Posteromedial (cm)	96.61 (14.64)	92.04 (13.26)	745.5	0.272	-0.151
Posterolateral (cm)	84.56 (13.79)	84.21 (15.58)	671	0.795	-0.036

Summary statistics are presented as *Mean (SD)*. Significance is indicated by bold text. r_rb_ = rank-biserial correlation

**Table 3:** Patient-Reported function scores

Variables	FAIS	DDH	U	*p*	*r*_rb_
Percent of Normal Function (%)	50.00 (40.00–70.00)	50.00 (35.00–60.00)	702	0.540	-0.084
UCLA Activity	6.00 (4.50–9.50)	4.00 (4.00–6.00)	845	**0.025**	-0.305
HOS ADL	76.32 (64.84–89.47)	65.79 (51.32–75.00)	868.5	**0.013**	-0.341
HOS Sport	45.72 (37.5–63.89)	45.00 (36.11–61.11)	700.5	0.554	-0.082
SF12 Physical Function	50.00 (25.00–75.00)	50.00 (25.00–50.00)	848.5	**0.021**	-0.310
HOOS ADL	72.50 (58.75–91.00)	67.00 (52.50–75.00)	804.5	0.078	-0.242
HOOS Sport	43.00 (31.00–62.00)	34.00 (23.25–43.00)	839.5	**0.030**	-0.297

Summary statistics are presented as *Median (IQR)*. Significance is indicated by bold text. r_rb_ = rank-biserial correlation

**Table 4:** Patient-reported pain scores

Variables	FAIS	DDH	U	*p*	*r*_rb_
HOOS Pain	62.00 (49.50–78.50)	53.00 (38.00–58.00)	425	**0.012**	0.344
Visual Analog Scale					
Average	4.00 (2.00–6.00)	6.00 (4.00–7.00)	478	0.054	0.262
Best	2.00 (1.00–3.00)	3.00 (2.00–4.00)	480.5	0.057	0.258
Worst	8.00 (6.50–9.00)	9.00 (8.00–9.00)	491	0.072	0.242
Pain Location Scale^a^					
Groin	3.00 (0.00–4.00)	3.00 (2.00–4.00)	578	0.431	0.107
Anterior Thigh	0.00 (0.00–1.00)	0.00 (0.00–2.00)	565.5	0.273	0.127
Knee	0.00 (0.00–2.00)	0.00 (0.00–3.00)	632	0.849	0.024
Low Back	2.00 (0.00–4.00)	2.00 (0.00–4.00)	545.5	0.468	0.097
Buttock	0.00 (0.00–2.50)	0.00 (0.00–1.00)	708	0.450	-0.093
Posterior Thigh	0.00 (0.00–0.00)	0.00 (0.00–0.00)	545.5	0.116	0.158
Trochanteric	2.00 (0.00–4.00)	3.00 (0.00–4.00)	597	0.568	0.078
Lateral Thigh	0.00 (0.00–0.00)	0.00 (0.00–0.00)	618	0.688	0.046

Summary statistics are presented as *Median (IQR)*. Lower HOOS Pain score indicates higher pain. Significance is indicated by bold text. Lower HOOS Pain score corresponds to higher pain. r_rb_ = rank-biserial correlation

^a^Pain location scale values are reported as the multiplication of frequency and intensity of pain scores

### Relationship between perceived and objectively measured functional ability

**Fig. 1:**
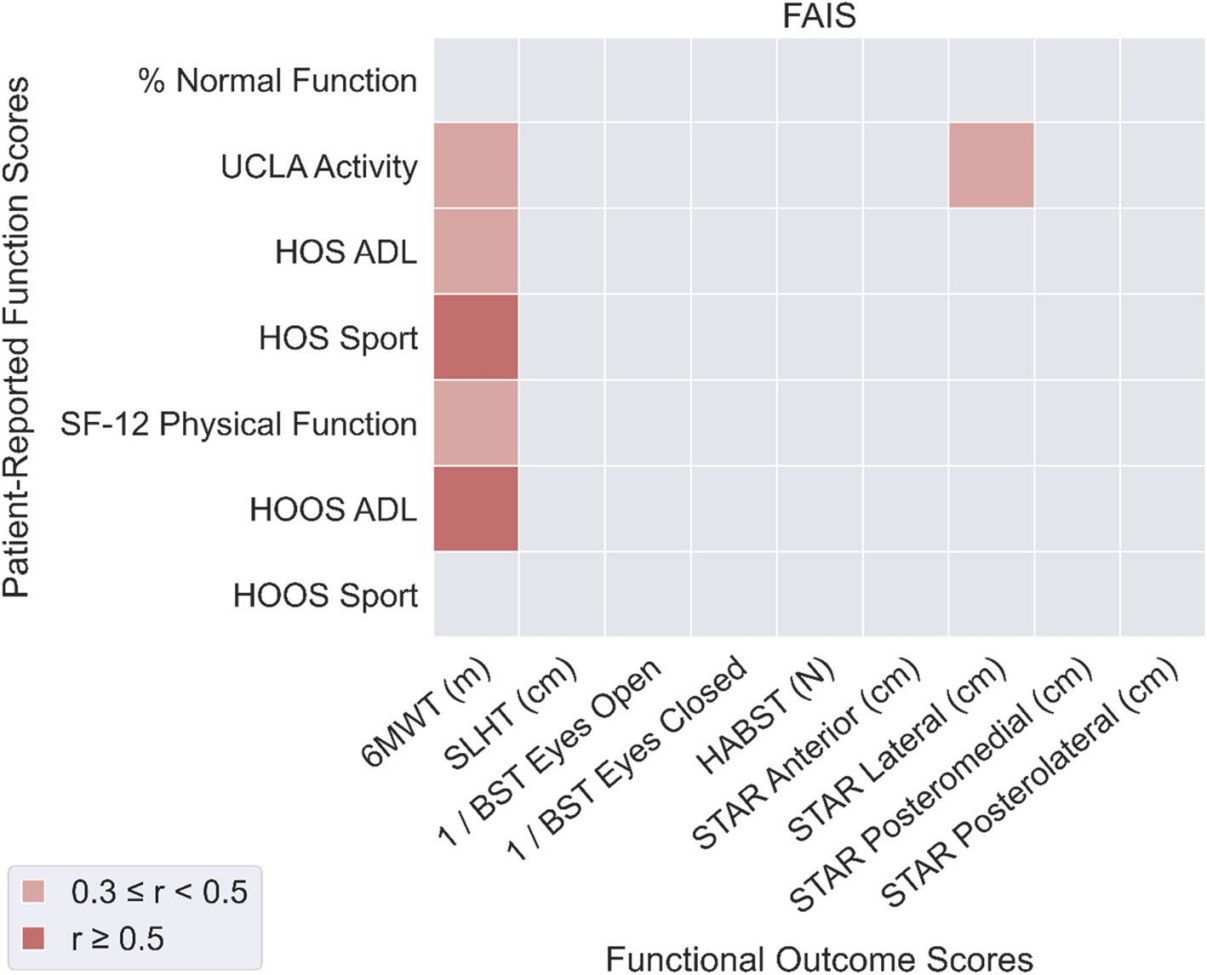
Correlations between functional outcomes (horizontal axis) and patient-reported function (vertical axis) for the FAI syndrome patient group

**Fig. 2:**
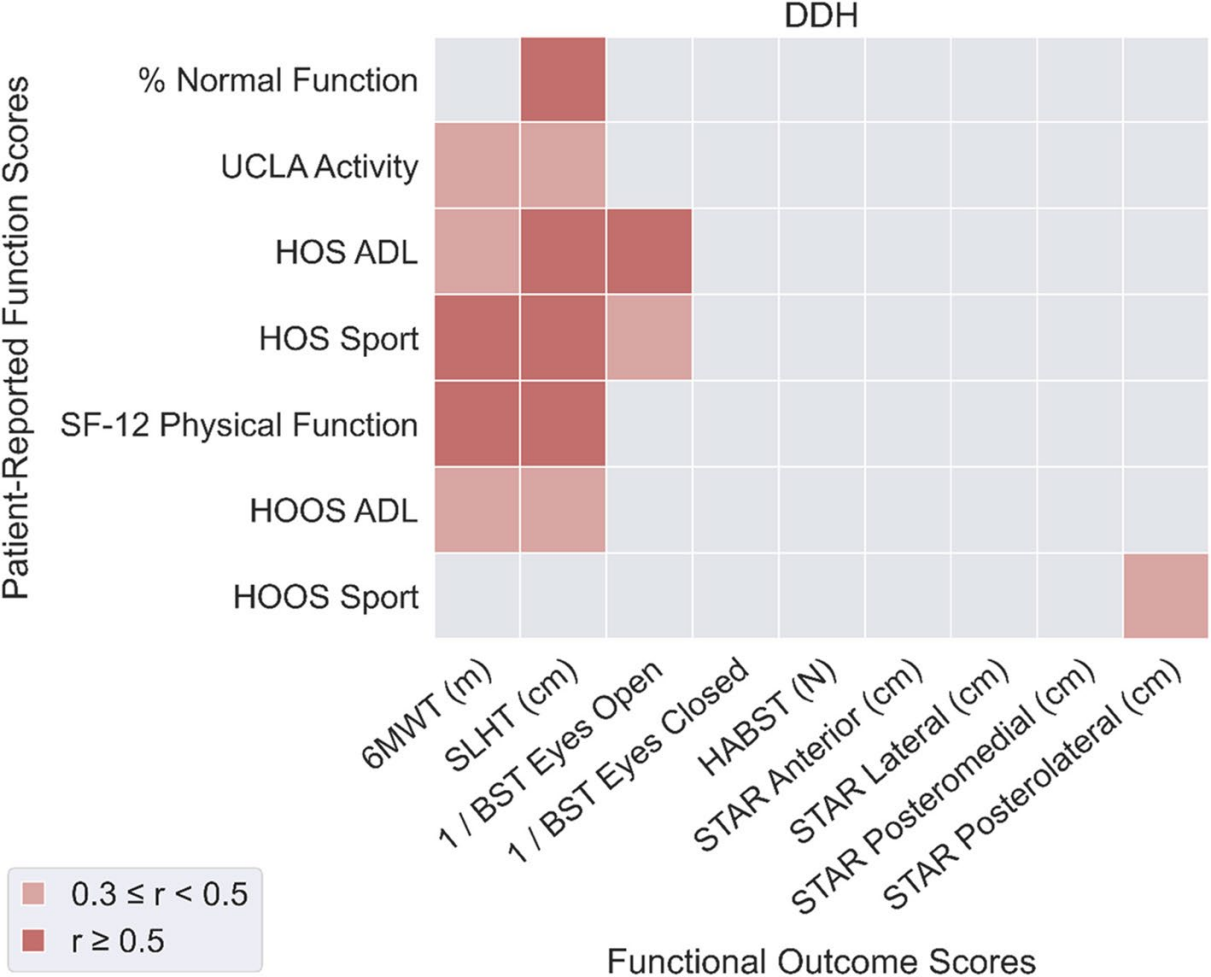
Correlations between functional outcomes (horizontal axis) and patient-reported function (vertical axis) for the DDH patient group

For both patient groups (FAIS: Fig. 1; DDH: Fig. 2), 6MWT distance was significantly correlated with PROM-F except for % Normal Function and HOOS Sport scores (*r* = 0.36 to 0.62).

In patients with DDH, SLHT distance was significantly correlated with all PROM-F measures except for HOOS Sport. BST scores were not correlated with PROM-F in patients with FAIS, but BST Eyes Open was well correlated with HOS ADL (*r* = 0.55) and Sport (*r* = 0.47) scores in patients with DDH. HABST score was not correlated with PROM-F in either patient group. Mean of all correlation coefficients was smaller for patients with FAIS (mean: 0.19, SD: 0.14) compared to patients with DDH (mean: 0.28, SD: 0.15), suggesting a weaker overall relationship between OBJ-F and PROM-F in patients with FAIS.

### Relationship between objectively measured functional ability and pain

**Fig. 3:**
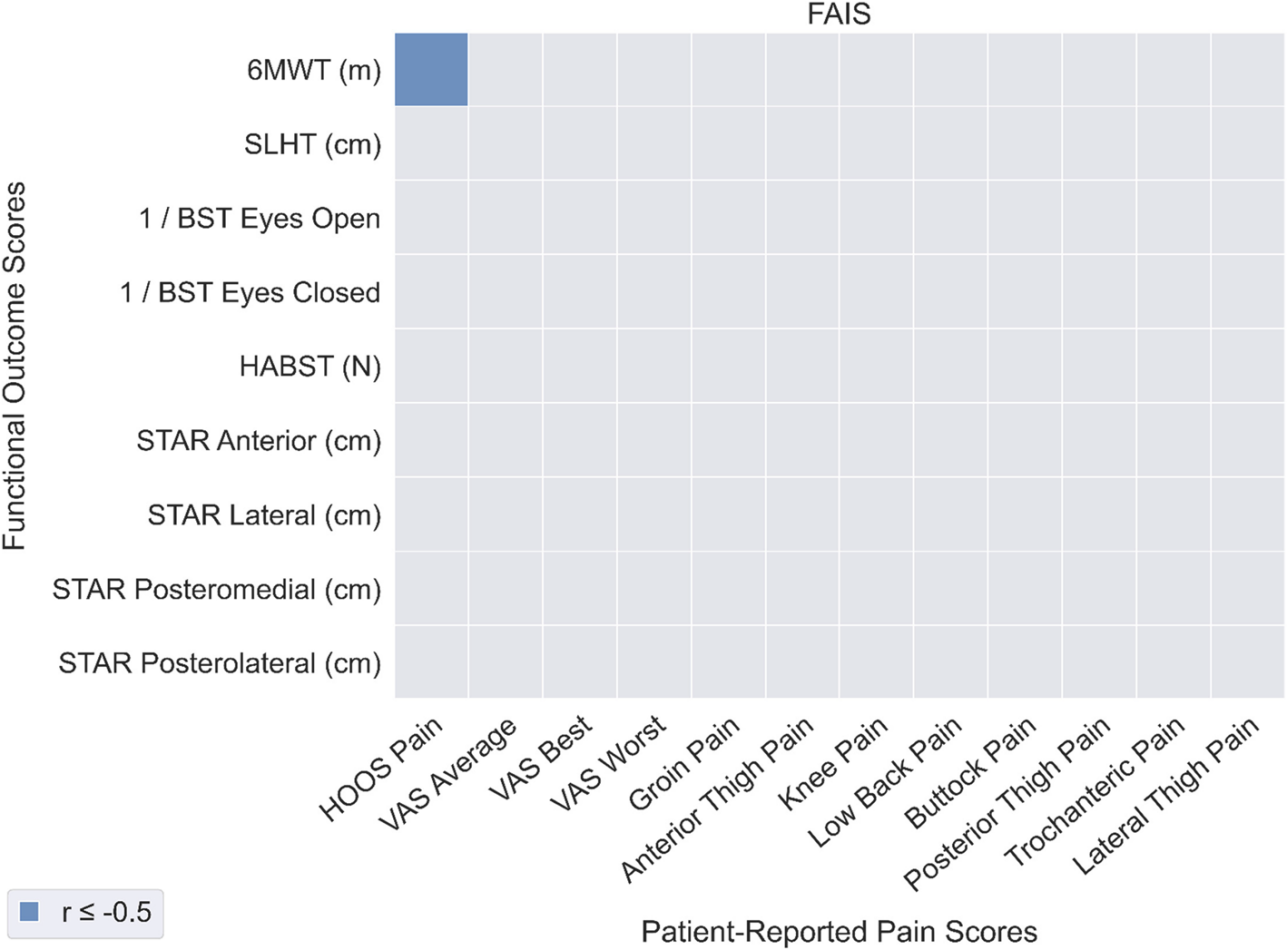
Correlations between patient-reported pain (horizontal axis) and functional outcomes (vertical axis) for the FAI syndrome patient group

**Fig. 4:**
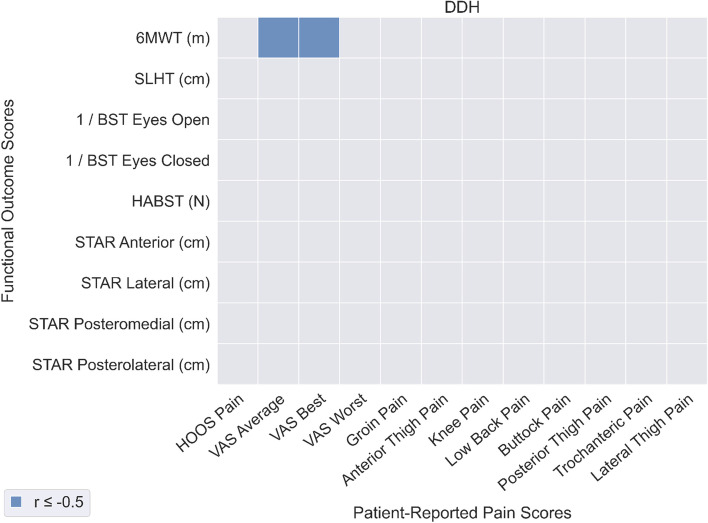
Correlations between patient-reported pain (horizontal axis) and functional outcomes (vertical axis) for the DDH patient group

In patients with FAIS (Fig. 3), the only significant correlation between PROM-P and OBJ-F was between 6MWT distance and HOOS Pain score (*r* = -0.53). In patients with DDH (Fig. 4), 6MWT distance was significantly correlated with VAS Average pain (*r* = -0.53) and VAS Best pain (*r* = -0.52). Overall, correlation coefficients for OBJ-F and PROM-P were low for both patients with FAIS (mean: 0.13, SD: 0.10) and DDH (mean: -0.05, SD: 0.19), indicating a weak relationship overall between OBJ-F and PROM-P in the two patient groups.

### Relationship between perceived functional ability and pain

**Fig. 5:**
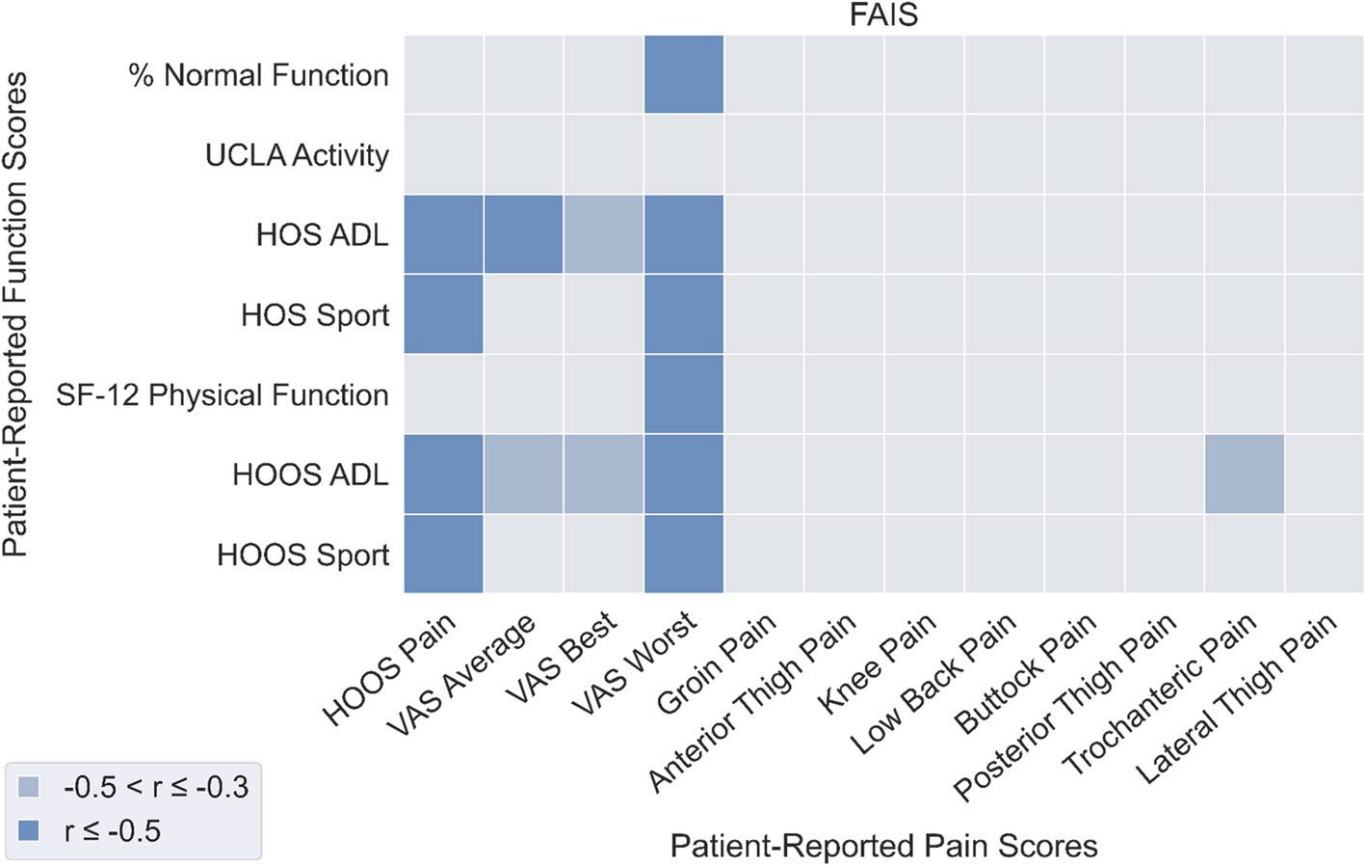
Correlations between patient-reported pain (horizontal axis) and patient-reported function (vertical axis) for the FAI syndrome patient group

**Fig. 6:**
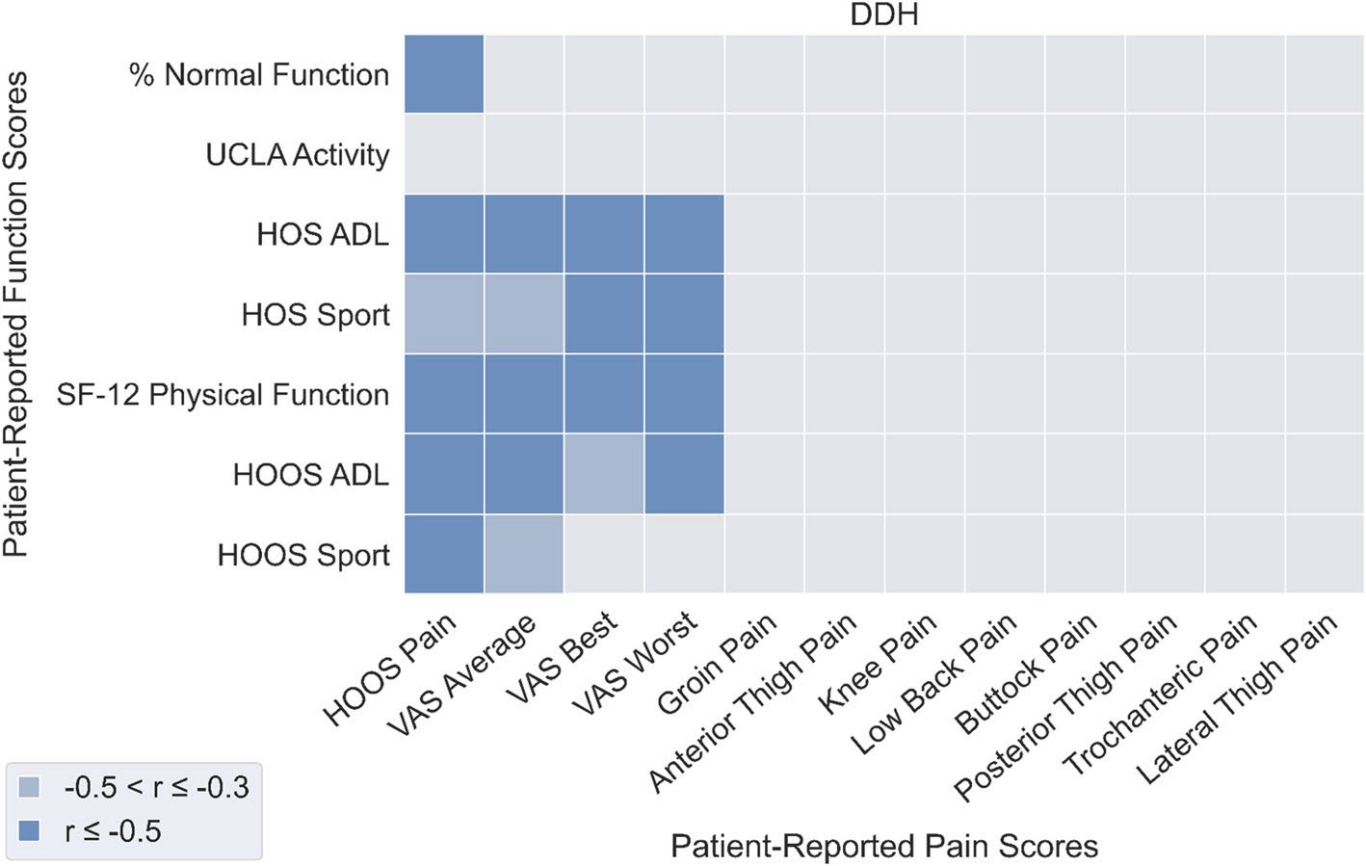
Correlations between patient-reported pain (horizontal axis) and patient-reported function (vertical axis) for the DDH patient group

Significant correlations between PROM-P and PROM-F were fewer in patients with FAIS (Fig. 5) compared to patients with DDH (Fig. 6). In patients with FAIS, a number of PROM-F measures were strongly significantly correlated with HOOS Pain (*r* = -0.58 to -0.93) and VAS Worst pain (*r* = -0.54 to -0.68).In patients with DDH, a number of PROM-F measures were significantly correlated with HOOS Pain (*r* = -0.45 to -0.82), and VAS Average (*r* = -0.47 to -0.72), Best (*r* = -0.45 to -0.62), and Worst (*r* = -0.53 to -0.65). PROM-P was overall better correlated to PROM-F than OBJ-F in both FAIS (mean: -0.24, SD: 0.21) and DDH (mean: -0.24, SD: 0.24) patients.

## Discussion

The purpose of this study was to evaluate differences of function and pain in patients with FAIS and patients with DDH, to assess the correlation between perceived and true functional ability, and to determine the influence of pain on measures of function. Delineating the symptoms of FAIS and DDH using common clinical measures could help facilitate accurate diagnosis of hip disease [40, 41] and shed new light on how self-perception of pain and function, and their relationships with objectively measured function may depend on underlying pathology.

Our first hypothesis, that patients with DDH would report higher pain and perform worse on functional tasks that demand hip power and stability compared to patients with FAIS, was partially supported. Pain levels were lower in patients with FAIS as indicated by HOOS Pain scores, but functional tasks with specific demands for hip power, balance, and stability—namely SLHT, BST, and STAR—were not significantly different between patient groups. Walking ability and hip abduction strength were significantly lesser in patients with DDH compared to patients with FAIS. Consistent with our findings, hip abduction strength decrements are well documented in patients with DDH [13, 14, 42]. Between-group differences in scores for UCLA Activity, HOS ADL, SF-12 Physical Function, and HOOS Sport suggest that patients with FAIS are able to participate in higher intensity physical activity with less interference from hip pain compared to patients with DDH. While attempting to distinguish FAIS and DDH through clinical measures, Kappe et al [40] found no significant differences in Western Ontario and McMaster Universities Arthritis Index (WOMAC) Pain and Function subscales between patient groups. WOMAC is comparable to HOS and HOOS.

Interestingly in our study, although HOS ADL and HOOS Sport subscales were significantly different between patient groups, HOS Sport and HOOS ADL subscales were not. The inclusion of relatively nondemanding tasks in HOS Sport and HOOS ADL may have contributed to their lack of differences. When evaluating patients with FAIS and patients with DDH separately, some studies have reported different PROM scores compared to our study. Wasko et al [43] reported higher mean HOOS ADL scores (mean: 66.6, SD: 21.6), higher HOOS Sport (mean: 40.9, SD: 25.3), and lower HOOS Pain (mean: 54.4, SD: 20.7) scores for a cohort of 302 pre-operative patients with DDH (sex: 84% female, median age: 21 [17 to 29] yrs, median BMI: 23.4 [20.8 to 26.3] kg/m^2^). Nepple et al [44] reported lower mean HOOS ADL (mean: 65.6, SD: 21.3), similar mean HOOS Sport (mean: 45.2, SD: 24.6), and lower HOOS Pain (mean: 56.1, SD: 20.7) for a cohort of 621 pre-operative patients with FAIS (sex: 50% female, mean age: 24.9 ± 9.1). These differences in reported symptoms illustrate the high variability and overlap of symptoms in FAIS and DDH. Simple comparisons of individual scores do not provide insight into how expressions of pain and function are dictated by the underlying hip pathology. Thus, we performed a detailed correlation analysis.

Our second hypothesis was that patients with FAIS would exhibit less widespread correlation between OBJ-F and PROM-F measures compared to patients with DDH. This hypothesis was supported, given the limited number of significant correlations for FAIS compared to DDH. In patients with FAIS, PROM-F instruments were well correlated only with walking ability. Lack of agreement between perceived and true function may point to the previously referenced protective mechanisms at play by these patients. In patients with DDH, PROM-F instruments were well correlated with walking ability as well as single leg hopping and postural stability, tasks known to be challenging for patients with DDH. Consistent with our findings, Scott et al [26] found strong correlations between common PROMs and functional tasks that have high demands for walking ability, lower limb strength, and dynamic balance in patients with DDH. Since 6MWT was well correlated with PROM-F in both patient groups and requires minimal time and equipment, the test could be performed in place of PROM-F instruments. Previous studies have examined correlations between the responsiveness of OBJ-F and PROM-F in post-operative total hip and knee replacement patients and found inconsistencies between objectively measured and perceived functional improvements [25, 28, 29]. Since the agreement among the responsiveness of these measurement modalities has not yet been studied in patients with pre-arthritic hip conditions, our findings serve as an important baseline for future study.

The UCLA Activity scale was previously validated by correlation with pedometry in lower limb joint reconstruction patients [45, 46]. However, latter items on the scale exceed the demands of normal walking. Thus, it was previously unclear how the scale relates to tasks that demand greater lower limb strength, power, postural stability, and dynamic balance. In the current study, UCLA Activity score was moderately positively correlated with objective measures of walking ability and dynamic balance in patients with FAIS, and with walking ability and single leg hopping in patients with DDH.

Our third hypothesis was that PROM-P would be well correlated with PROM-F, and only weakly correlated with OBJ-F in both patient groups. This hypothesis was well supported. Correlations between OBJ-F and PROM-P were sparse in both patient groups, as only one significant correlation was observed in patients with FAIS, and two were observed in patients with DDH. Unlike patients with other hip and knee pathologies, functional performance of patients with DDH is strongly influenced by pain [17, 19, 21–24, 28]. Overall PROM-P measures had better agreement with PROM-F than with OBJ-F measures. This suggests that pain has a stronger influence on perceived function than true function [18, 20].

Some limitations were present in our study. Because all included patients were scheduled for preservative surgery, they represent the most symptomatic patients of their respective pathology. Thus, application of our findings should be limited to patients who are moderately-to-severely symptomatic. Secondly, our findings may have been influenced by the differing age and sex distribution between FAIS and DDH. Nevertheless, our patient sample were a consecutive series of pre-operative patients from our hip preservation practice, and their age and sex distributions reflect those of typical demographics for these pathologies [47, 48].

Future work should investigate the relationship between post-operative improvements in PROMs and OBJ-F in patients with FAIS and patients with DDH. Additionally, a study that includes non-operative patients with FAIS and DDH may provide a broader view of how hip morphology influences pain and function. Furthermore, as suggested by Hampton et al [49]_,_ it may be possible to alter how patients perceive their functional ability and experience of pain to promote further recovery of function post-operatively.

## Conclusions

Our study highlights that patients with DDH experience increased pain and decreased function compared to patients with FAIS. Moreover, the relationships between pain and function appear to differ depending on the underlying hip pathology. This was notably reflected in the dissimilar correlations between patient-reported pain measures (i.e., HOOS Pain and VAS) with patient-reported function measures. Understanding these differences is valuable for informing clinical management and treatment decisions. We recommend that these insights should be incorporated in the evaluation and selection of surgical and therapeutic interventions by prospective patients. Finally, our study revealed a close correlation between performance during sustained walking bouts (i.e., 6MWT) and patient-reported pain and function in both patient cohorts. In patients with DDH specifically, we demonstrated an additional correlation between performance during hopping (i.e., SLHT) and patient-reported function. Therefore, we recommend that clinicians could utilize these practical functional measures, especially when the administration of multiple patient-reported outcome measures may be infeasible or burdensome to acquire within the clinical setting.

### Supplementary Information

**Additional file 1.**

**Additional file 2. **

**Additional file 3. **

**Additional file 4. **

**Additional file 5. **

**Additional file 6. **

## Data Availability

The datasets used and/or analyzed during the current study are available from the corresponding author on reasonable request.

## Abbreviations

6MWT: 6-Minute walk test
ADL: Activities of daily living
BST: Biodex sway test
DDH: Developmental dysplasia of the hip
FAIS: Femoroacetabular impingement syndrome
HABST: Hip abduction strength test
HOS: Hip Outcome Score
HOOS: Hip Disability and Osteoarthritis Outcome Score
OBJ-F: Objective measures of function
PROM-F: Patient-reported measures of function
PROM-P: Patient-reported measures of pain
ROM: Range of motion
SF-12: Short Form 12
SLHT: Single leg hop test
STAR: Star excursion balance reach test
VAS: Visual analogue scale
